# Synergistic use of 1,5-AG and HbA1c for early prediction of gestational diabetes: capturing BMI-dependent glycemic phenotypes

**DOI:** 10.1007/s00404-025-08281-3

**Published:** 2026-01-02

**Authors:** Sho Tano, Tatsuo Inamura, Kazuya Fuma, Seiko Matsuo, Kenji Imai, Satoru Katsuki, Yasuyuki Kishigami, Hidenori Oguchi, Tomomi Kotani, Takafumi Ushida, Hiroaki Kajiyama

**Affiliations:** 1 Department of Obstetrics and Gynecology, Nagoya University Graduate School of Medicine, Showa-Ku Tsurumai 65, Nagoya, Aichi 466-8560 Japan; 2 Department of Obstetrics, Perinatal Medical Center, Toyota Memorial Hospital, Toyota, Aichi 471-8513 Japan; 3 Department of Obstetrics and Gynecology, Hamamatsu University School of Medicine, Hamamatsu, Shizuoka 431-3192 Japan

**Keywords:** 1,5-Anhydroglucitol, Glycemic variability, Pre-pregnancy BMI, Predictive model, Postprandial hyperglycemia

## Abstract

**Purpose:**

Recognizing metabolic heterogeneity in gestational diabetes mellitus (GDM) and body mass index (BMI)-linked phenotypes, we evaluated whether combining hemoglobin A1c (HbA1c, reflecting fasting glycaemia) and 1,5-anhydroglucitol (1,5-AG, reflecting post-load glucose excursions) improves early prediction and whether performance differs by BMI.

**Methods:**

In this multicenter retrospective study, pregnant women who had 1,5-AG and HbA1c measured before 20 weeks of gestation at two tertiary centers in Japan were included. Spearman’s correlation was used to assess associations between glycemic markers and glucose levels. Predictive performance for GDM was evaluated using ROC analysis, and stratified analyses were conducted by pre-pregnancy BMI.

**Results:**

Among 191 participants, 45 (24.1%) developed GDM: 35.1 ± 4.9 years, pre-pregnancy BMI 22.9 ± 4.3 kg/m^2^, and sampling at 14.3 [IQR 14.0–14.7] weeks. HbA1c correlated with fasting glucose (*r* = 0.35) while 1,5-AG correlated inversely with 2-h glucose (*r* =  − 0.39). They themselves were not significantly correlated (*r* =  − 0.13). As single predictors, performance depended on BMI: in ≥ 25.0 kg/m^2^, HbA1c outperformed 1,5-AG (AUC 0.776 vs 0.618); in BMI < 25.0 kg/m^2^, 1,5-AG outperformed HbA1c (AUC 0.704 vs 0.640). In both BMI strata, the dual-marker model was superior (AUC 0.833 and 0.803, respectively) and provided more balanced sensitivity, accuracy, and F1. Pre-pregnancy BMI correlated positively with fasting plasma glucose (*r* = 0.47) but not with 1-h or 2-h glucose (*r* = 0.20 and *r* = 0.16, respectively), supporting BMI-related metabolic variation.

**Conclusion:**

Combining 1,5-AG and HbA1c enhances early prediction of GDM by capturing complementary glycemic abnormalities linked to BMI-specific metabolic phenotypes.

**Supplementary Information:**

The online version contains supplementary material available at 10.1007/s00404-025-08281-3.

## Introduction

Gestational diabetes mellitus (GDM) and impaired glucose metabolism during pregnancy are associated with adverse maternal and fetal outcomes, including an increased risk of short- and long-term metabolic disorders in mothers and children [1–4]. According to current diagnostic criteria, GDM is identified when either fasting plasma glucose (FPG) or post-load glucose levels during an oral glucose tolerance test (OGTT) exceed defined thresholds [5, 6]. As a result, GDM encompasses a spectrum of pathophysiologically distinct phenotypes under a single diagnostic label. In other words, women with elevated fasting glucose and those with postprandial hyperglycemia are classified as having the same disorder despite differing underlying mechanisms of dysglycemia.

Obesity is well known to be associated with elevated fasting glucose levels due to increased hepatic glucose production and insulin resistance [7–9]. In contrast, exaggerated post-load glucose excursions are often driven by impaired early-phase insulin secretion and may reflect underlying genetic susceptibility influencing β‑cell function. [10, 11] These observations highlight the heterogeneity of gestational glucose metabolism, with differing mechanisms of hyperglycemia across individuals.

Given this heterogeneity, we consider that it is insufficient to rely on a single biomarker to predict GDM risk. Hemoglobin A1c (HbA1c) primarily reflects mean plasma glucose levels [12], while 1,5-anhydroglucitol (1,5-AG) is a validated marker of short-term glycemic excursions, particularly postprandial spikes [13, 14]. We hypothesized that combining these two complementary markers may enhance early prediction of GDM by capturing the diverse glycemic profiles associated with body mass index (BMI)-dependent metabolic phenotypes. In this study, we evaluated the predictive performance of 1,5-AG and HbA1c—both individually and in combination—across different BMI strata.

## Methods

### Study design and participants

This retrospective study was conducted at two centers (Nagoya University Hospital and TOYOTA Memorial Hospital) from December 2022 to December 2024. The datasets used comprised electronic hospital records of pregnant women aged 18 years or older. Pregnant women who voluntarily underwent measurements of 1,5-AG and HbA1c levels before 20 weeks of gestation were included in the analysis. Women were excluded if they were prepregnant or had overt diabetes mellitus (DM).

This study was approved by the ethics board of Nagoya University Hospital (approval number: 2015–0415, registered on August 4, 2022) and was conducted in accordance with the Declaration of Helsinki. The requirement for informed consent was waived because of the retrospective nature of the study.

### Glucose assessment and 75 g OGTT

In accordance with Japanese clinical guidelines [5], pregnant women with random blood glucose (RBG) levels exceeding 100 mg/dL (5.6 mmol/L) underwent a 75 g OGTT. The initial screening was performed in early pregnancy at a median of 14.3 weeks (IQR 14.0–14.7). In the OGTT, plasma glucose levels were measured at FPG and at 1 h and 2 h after glucose loading (1 h-PG and 2 h-PG, respectively). GDM was diagnosed if at least one of the following thresholds were met: FPG ≥ 92 mg/dL (5.1 mmol/L), 1 h-PG ≥ 180 mg/dL (10.0 mmol/L), or 2 h-PG ≥ 153 mg/dL (8.5 mmol/L). For individuals who tested negative in the first screening, the second screening was conducted at approximately 24–28 weeks of gestation using RBG with the same cutoff value or a 50 g glucose challenge test (GCT). A 1-h PG level of ≥ 140 mg/dL (7.8 mmol/L) following GCT prompted a follow-up 75 g OGTT using the same cutoff value. Both institutions used the same diagnostic protocols.

### Definitions of variables

Overt-DM was defined as an HbA1c ≥ 6.5% (48 mmol/mol) or an FPG ≥ 126 mg/dL (7.0 mmol/L) during pregnancy [5]. Maternal pre-pregnancy body weight, self-reported during routine practice, was used to calculate the pre-pregnancy body mass index (BMI, kg/m^2^). Overweight/obesity was defined as a pre-pregnancy BMI of ≥ 25.0 kg/m^2^. Gestational age was determined on the basis of the last menstrual period or crown–rump length measurement during routine practice [5]. Serum 1,5-AG was measured by an external accredited reference laboratory under routine quality control procedures in both facilities.

### Statistical analysis

The normality of continuous variables was assessed via the Shapiro‒Wilk analysis. For comparisons of baseline characteristics, the t test was used for normally distributed variables, and the Mann–Whitney *U* test was applied for non-normally distributed variables. Categorical variables were compared via the chi-square test or Fisher’s exact test, as appropriate. The correlations among RBG, HbA1c, 1,5-AG, and OGTT-derived glucose levels were analyzed. Spearman’s correlation analysis was used to evaluate these associations.

The primary analysis included receiver operating characteristic (ROC) curve analysis to evaluate the diagnostic performance of RBG, 1,5-AG, and HbA1c levels for predicting GDM. In addition to single-predictor analyses, multivariable logistic regression models incorporating combinations of predictors (1,5-AG + HbA1c) were developed to evaluate the diagnostic performance of combined predictors. DeLong tests compared the combined model with single markers. Cut-off values were selected by Youden index (YI). The sensitivity, specificity, accuracy, positive predictive value (PPV), and negative predictive value (NPV) were calculated to evaluate performance efficacy. We also reported the F1 score (the harmonic mean of precision and recall) to complement ROC-based metrics under class imbalance. To visualize the distribution patterns of 1,5-AG and HbA1c across GDM groups, bivariate scatterplots with overlaid 95% confidence ellipses were generated via the multivariate t distribution assumption. [15] Furthermore, a multivariate analysis of variance (MANOVA) was conducted to assess whether the joint distribution of 1,5-AG and HbA1c levels significantly differed between women with and without GDM [16]. Wilks’ lambda was used as a test statistic to evaluate group effects [16]. Given that previous studies have reported an association between 1,5-AG and overweight/obesity [17, 18], additional stratified analyses were conducted on the basis of overweight/obesity status.

Statistical significance was set at a two-tailed *p* value < 0.05. Statistical analyses, including model development, performance evaluation, and multivariate regression analysis, were conducted via R software, version 4.1.3 (https://cran.r-project.org/).

## Results

A total of 191 pregnant women who voluntarily underwent measurements of 1,5-AG and HbA1c levels before 20 weeks of gestation were initially identified. After four individuals with pre-pregnancy DM (*n* = 2) or overt DM (*n* = 2) were excluded, 187 pregnancies were included in the final analysis (Figure S1). All participants completed the second screening by March 2025, and no patients were excluded because of miscarriage or other causes of loss to follow-up. Table 1 summarizes the baseline characteristics and biochemical profiles stratified by pre-pregnancy BMI category; 43 patients were categorized as overweight/obese, and 144 patients were classified as non-overweight/obese. Gestational age at measuring RBG, HbA1c and 1,5-AG was approximately 14.3 weeks of gestation (IQR 14.0–14.7 weeks). The incidence of GDM was greater in the overweight/obese group than in the non-overweight/obese group (41.9% vs. 18.8%, *p* = 0.007). No significant differences were observed in maternal age, parity, or gestational age at testing between the two groups. Although the HbA1c levels in the overweight/obese and non-overweight/obese groups were 5.5% and 5.4%, respectively, FPG differed significantly between the groups (90.0 vs. 86.0 mg/dL, *p* = 0.004). Although not statistically significant, both the 1-h and 2-h post-load glucose levels tended to be higher in the overweight/obese group than in the non-overweight/obese group (1 h-PG: 169.0 vs. 153.0 mg/dL, *p* = 0.140; 2 h-PG: 142.0 vs. 134.0 mg/dL, *p* = 0.348). Similarly, 1,5-AG levels were higher in the overweight/obese group than in the non-overweight/obese group, although the difference was statistically insignificant (13.4 vs. 10.2 μg/mL, *p* = 0.110).

**Table 1:** Baseline characteristics of the study population

	Overall (*n* = 187)	Overweight/obesity (*n* = 43)	Non-overweight/obesity (*n* = 144)	*p* value
Age, years	35.1 ± 4.9	34.3 ± 5.7	35.3 ± 4.5	0.309
Pre-pregnancy BMI, kg/m^2^	22.9 ± 4.3	29.7 ± 4.6	20.8 ± 1.9	< 0.001*
Primiparity	93 (49.7)	23 (53.5)	70 (48.6)	0.938
Gestational age at test, weeks	14.3 [14.0, 14.7]	14.4 [14.3, 14.7]	14.3 [14.0, 14.7]	0.837
HbA1c, %	5.5 [5.3, 5.6]	5.5 [5.3, 5.7]	5.4 [5.2, 5.5]	0.002*
1,5-AG, μg/dL	10.1 [7.6, 13.7]	13.4 [8.1, 16.5]	10.2 [7.9, 13.7]	0.110
RBG, mg/dL	91 [85, 105]	97 [90, 111]	89 [84, 101]	0.004*
OGTT performed (RBG ≥ 100 mg/dL)	57 (30.5)	19 (44.2)	38 (26.4)	0.030*
FPG^†^, mg/dL	89 [84, 92]	90 [88, 97]	86 [83, 90]	0.004*
1 h-PG^†^, mg/dL	114 [106, 128]	169 [143, 185]	153 [136, 166]	0.140
2 h-PG^†^, mg/dL	135 [119, 157]	142 [120, 159]	134 [120, 152]	0.348
GDM, *n* (%)	45 (24.1)	18 (41.9)	27 (18.8)	0.007*

The values are presented as the means ± standard deviations or medians [Q_1_, Q_3_] for continuous variables and as numbers (%) for categorical variables. *p* values indicate comparisons between the overweight/obese and non-overweight/obese groups

*BMI* body mass index, *HbA1c* hemoglobin A1c, *1,5-AG* 1,5-anhydroglucitol, *RBG* random blood glucose, *FPG* fasting plasma glucose, *1 h-PG* 1 h plasma glucose levels after loading, *2 h-PG* 2 h plasma glucose levels after loading, *GDM* gestational diabetes mellitus

^*^Statistically significant (*p* < 0.05)

^†^FPG, 1 h-PG, and 2 h-PG were derived from a 75 g OGTT conducted at 20 weeks of gestation in participants with RBG ≥ 100 mg/dL

**Fig. 1:**
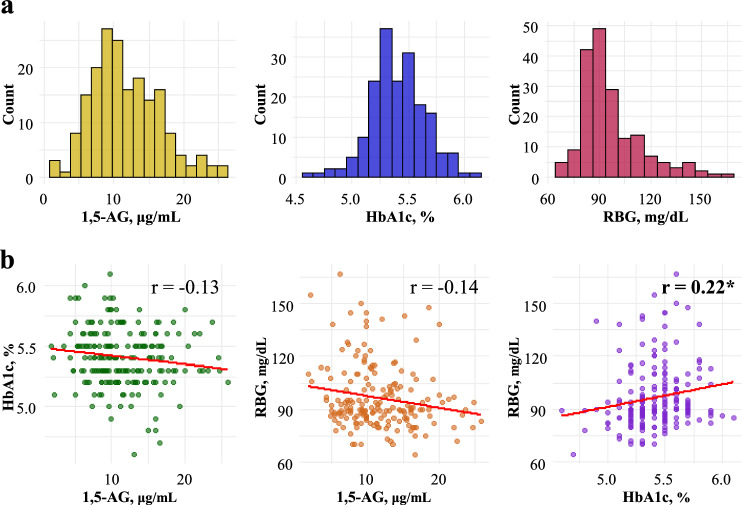
Distributions and correlations of early pregnancy biomarkers. **a** Histograms showing the frequency distributions of 1,5-anhydroglucitol (1,5-AG), hemoglobin A1c (HbA1c), and random blood glucose (RBG) levels measured before 20 weeks of gestation. **b** Scatter plots (bottom row) illustrating Spearman’s rank correlation coefficients (*r*) between each biomarker pair

As shown in Fig. 1a, the distribution of 1,5-AG ranged from 1.8 to 25.9 μg/mL, whereas that of HbA1c ranged from 4.6% to 6.1%. The RBG levels varied from 64 mg/dL to 167 mg/dL. Figure 1b shows pairwise correlations among these biomarkers. Overall, no strong correlations were observed among these markers. The correlation coefficients were weak, indicating minimal associations between 1,5-AG and HbA1c (*r* = −0.13), between 1,5-AG and RBG (*r* = −0.14), and between HbA1c and RBG (*r* = 0.22).

**Fig. 2:**
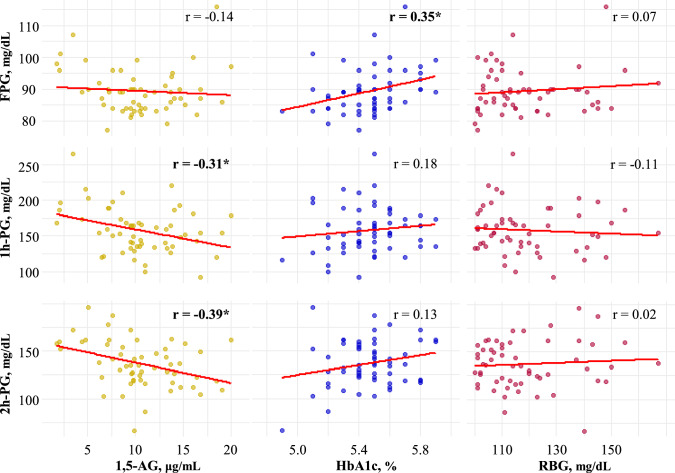
Correlations between early pregnancy biomarkers and clinical parameters. Scatter plots showing Spearman’s rank correlation coefficients (*r*) between each biomarker and OGTTs-derived glucose levels. *FPG* fasting plasma glucose, *1 h-PG* 1 h plasma glucose after loading, *2 h-PG* 2 h plasma glucose after loading, *RBG* random blood glucose

Figure 2 illustrates the correlations between biomarkers and OGTT results in participants with RBG ≥ 100 mg/dL (*n* = 57). The value of 1,5-AG (left panels) was significantly and negatively correlated with both 1 h-PG (*r* = −0.31) and 2 h-PG (*r* = −0.39) but not with FPG (*r* = −0.14). In contrast, HbA1c (middle panels) was moderately correlated with FPG (*r* = 0.35), whereas its correlations with 1 h-PG and 2 h-PG were weak or absent (*r* = 0.18 and 0.13, respectively). The RBG (right panels) did not demonstrate significant correlations with any of the OGTT parameters.

**Fig. 3:**
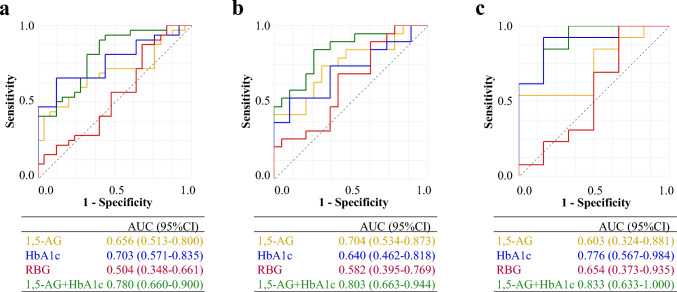
Diagnostic performance of biomarkers for GDM in the overall and BMI-stratified cohorts. ROC curves comparing the ability of 1,5-AG, HbA1c, RBG, and their combination (1,5-AG + HbA1c) to predict GDM. AUC values are shown, **a** Overall cohort; **b** non-overweight/obesity (BMI < 25.0 kg/m^2^); **c** Overweight/obesity (BMI ≥ 25.0 kg/m^2^)

Figure 3a shows the ROC curves for individual biomarkers in predicting GDM. Among the single predictors, HbA1c had the highest performance (AUC = 0.703), followed by 1,5-AG (AUC = 0.656), whereas RBG had poor discriminative ability (AUC = 0.496). The combined model of 1,5-AG and HbA1c significantly improved AUC of 0.780. According to the logistic regression model, both biomarkers were independently associated with GDM risk (β for 1,5-AG = –0.195, β for HbA1c = 5.222; intercept = −26.165). In women with a BMI < 25.0 kg/m^2^, 1,5-AG demonstrated the best performance among the individual markers (AUC = 0.704), whereas the combination model further improved the prediction (AUC = 0.803; Fig. 3b). Among women with a BMI ≥ 25.0 kg/m^2^ (Fig. 3c), HbA1c alone had the highest individual performance (AUC = 0.776), whereas the combined model again provided the best overall prediction (AUC = 0.833). As shown in Table 2, DeLong tests comparing each single marker against the combined model (HbA1c + 1,5-AG) indicated significant AUC differences for RBG (overall *p* = 0.004; BMI < 25.0 kg/m^2^ *p* = 0.028; BMI ≥ 25.0 kg/m^2^ *p* = 0.001) and for 1,5-AG in the overall cohort (*p* = 0.045) and HbA1c in BMI < 25.0 kg/m^2^ (*p* = 0.044), with other contrasts not reaching significance.

**Table 2:** Diagnostic performance of individual biomarkers and the combined model

	AUC (95%CI)	*p* value* (DeLong)	cut-off	Sensitivity (95%CI)	Specificity (95%CI)	Accuracy	PPV	NPV	F1
Overall cohort									
1,5-AG	0.656 (0.513–0.800)	0.045*	8.00	0.41 (0.24–0.59)	0.92 (0.74–0.99)	0.80	0.87	0.55	0.55
HbA1c	0.703 (0.571–0.835)	0.163	5.55	0.47 (0.29–0.65)	0.88 (0.69–0.98)	0.78	0.83	0.56	0.60
RBG	0.504 (0.348–0.661)	0.004*	119	0.31 (0.16–0.50)	0.56 (0.35–0.76)	0.50	0.48	0.39	0.38
1,5-AG + HbA1c	0.780 (0.660–0.900)	–	0.40	0.91 (0.75–0.98)	0.56 (0.35–0.76)	0.64	0.73	0.82	0.81
BMI < 25.0 kg/m^2^									
1,5-AG	0.704 (0.534–0.873)	0.083	8.00	0.42 (0.20–0.67)	0.95 (0.74–1.00)	0.85	0.62	0.62	0.57
HbA1c	0.640 (0.462–0.818)	0.044*	5.55	0.37 (0.16–0.62)	0.90 (0.67–0.99)	0.80	0.59	0.58	0.50
RBG	0.582 (0.395–0.769)	0.028*	119	0.37 (0.16–0.62)	0.58 (0.34–0.80)	0.54	0.48	0.48	0.41
1,5-AG + HbA1c	0.803 (0.663–0.944)	–	0.44	0.84 (0.60–0.97)	0.68 (0.41–0.87)	0.71	0.81	0.79	0.78
BMI ≥ 25.0 kg/m^2^									
1,5-AG	0.603 (0.324–0.881)	0.098	9.25	0.54 (0.25–0.81)	0.83 (0.36–1.00)	0.71	0.88	0.46	0.67
HbA1c	0.776 (0.567–0.984)	0.567	5.55	0.62 (0.32–0.86)	0.83 (0.36–1.00)	0.74	0.89	0.50	0.73
RBG	0.654 (0.373–0.935)	0.001*	114	0.31 (0.09–0.61)	0.33 (0.04–0.78)	0.32	0.50	0.18	0.38
1,5-AG + HbA1c	0.833 (0.633–1.000)	–	0.54	0.85 (0.55–0.98)	0.67 (0.22–0.96)	0.75	0.85	0.67	0.85

*PPV* positive predictive value, *NPV* negative predictive value, *BMI* body mass index, *HbA1c* hemoglobin A1c, *1,5-AG* 1,5-anhydroglucitol, *RBG* random blood glucose

^*^p value for paired DeLong test comparing the combined model (HbA1c + 1,5-AG) with each single marker within the same stratum. *p < 0.05

Table 2 revealed that on the basis of optimal cutoffs via the Youden index in the overall cohort. The combined model (1,5-AG + HbA1c) demonstrated the highest sensitivity and F1 score. While both HbA1c and 1,5-AG had relatively high specificity, RBG performed poorly across all the metrics. As shown in Fig. S2a, the distributions of 1,5-AG and HbA1c levels differed significantly by GDM status. MANOVA confirmed a significant multivariate group effect (Wilks’ lambda = 0.867, F[2,182] = 13.93, *p* < 0.001), and subsequent univariate analyses revealed that women with GDM had lower 1,5-AG (F[1,183] = 8.80, *p* = 0.003) and higher HbA1c levels (F[1,183] = 21.03, *p* < 0.001), supporting their individual contributions to GDM risk stratification.

In women with a BMI < 25.0 kg/m^2^, the combination model (1,5-AG + HbA1c) also demonstrated the highest sensitivity and F1 score (Table 2). As for single predictors, 1,5-AG had relatively good specificity, accuracy, and PPV. MANOVA again demonstrated a significant group effect (Wilks’ lambda = 0.867, F[2,139] = 10.65, *p* < 0.001), and both 1,5-AG (F[1,140] = 11.49, *p* = 0.001) and HbA1c (Figure S2b, F[1,140] = 11.96, *p* < 0.001) showed significant group differences. These findings suggest that the discriminatory value of 1,5-AG and HbA1c in relation to GDM persists even in non-overweight/obese women, highlighting their potential clinical utility beyond high BMI populations.

In women with a BMI ≥ 25.0 kg/m^2^, the combined model (1,5-AG + HbA1c) further improved the sensitivity and F1 score (Table 2). As for single predictors, HbA1c alone had relatively good specificity, accuracy, and PPV. MANOVA confirmed a significant but less pronounced group difference in biomarker profiles (Figure S2c, Wilks’ lambda = 0.855, F[2,40] = 3.38, *p* = 0.044). While HbA1c remained significantly elevated in women with GDM (F[1,41] = 5.67, *p* = 0.022), the difference in 1,5-AG was not statistically significant (F[1,41] = 1.82, *p* = 0.185). These findings suggest that although the overall multivariate difference between the GDM and non-GDM groups remained significant among women with higher BMIs, the contribution of 1,5-AG to group discrimination may be attenuated in this subgroup, whereas HbA1c retained its discriminatory value.

**Fig. 4:**
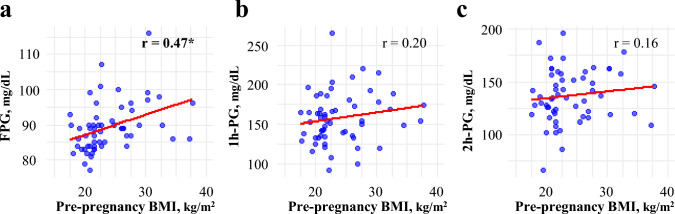
Correlation between pre-pregnancy BMI and OGTT glucose levels. Scatter plots showing the correlation between pre-pregnancy BMI and glucose values during the OGTT performed at 20 weeks of gestation: **a** fasting plasma glucose (FPG); **b** 1-h plasma glucose (1 h-PG); and **c** 2-h plasma glucose (2 h-PG). The red lines indicate the fitted linear regression lines. Spearman’s rank correlation coefficients (*r*) are shown in each panel

Figure 4 illustrates the exploratory analyses conducted to understand why the predictive performance of 1,5-AG and HbA1c differed according to pre-pregnancy BMI. As shown in Fig. 4a, pre-pregnancy BMI was significantly correlated with FPG (*r* = 0.47).

## Discussion

This study demonstrated that both 1,5-AG and HbA1c levels measured before 20 weeks of gestation were significantly associated with a subsequent GDM diagnosis. Although HbA1c showed a slightly greater overall diagnostic performance, 1,5-AG provided complementary information, particularly reflecting post-load glucose levels rather than fasting glucose levels. The combination of 1,5-AG and HbA1c had greater predictive accuracy than either marker alone. Furthermore, stratified analyses revealed that the predictive performance of these biomarkers differed according to pre-pregnancy BMI: 1,5-AG outperformed HbA1c in women with BMI < 25.0 kg/m^2^, whereas HbA1c performed better in those with BMI ≥ 25.0 kg/m^2^. Importantly, across all BMI categories, the combination of 1,5-AG and HbA1c consistently demonstrated superior diagnostic performance to either marker alone, underscoring the value of a combined biomarker approach for early risk stratification.

Another key finding was the differential predictive value of biomarkers depending on the pre-pregnancy BMI. In women with higher BMI, HbA1c showed modestly better discrimination than 1,5-AG, whereas in women with lower BMI, 1,5-AG tended to discriminate better than HbA1c. The pattern is compatible with, but does not establish, greater fasting-glycemia contribution in higher-BMI phenotypes and greater post-prandial contribution in lower-BMI phenotypes. Possible obesity-related influences on 1,5-AG are hypotheses that require prospective validation. Despite the higher glucose levels observed in overweight/obese women, 1,5-AG levels were also relatively high in this group. This paradoxical result suggests that 1,5-AG itself may be influenced by obesity-related factors [17, 18], such as altered renal glucose handling or baseline metabolic status. This effect may partly explain the attenuated predictive performance of 1,5-AG for GDM in overweight/obese individuals. However, these mechanisms remain speculative in our data; we did not directly measure renal tubular function, diet, or other intermediates that would be required to test these pathways. Accordingly, unmeasured factors such as kidney function, plasma-volume status, and dietary 1,5-AG intake may confound these associations, and prospective studies with targeted intermediate phenotyping are needed.

Previous studies have evaluated the utility of 1,5-AG and HbA1c levels individually for predicting GDM, with mixed findings. For example, Pramodkumar et al. reported that 1,5-AG is significantly associated with GDM and has a higher C statistic than does HbA1c alone [19]. Similarly, Saglam et al. demonstrated that both 1,5-AG and HbA1c levels were significantly different between GDM and non-GDM groups, although neither marker alone provided sufficient diagnostic accuracy [20]. Several studies report the limited utility of 1,5-AG for GDM diagnosis at 24–28 weeks (low specificity or only moderate AUCs), and some find no predictive value [19, 21, 22]. These studies largely sampled in mid-pregnancy. We note that pregnancy-specific physiology—including plasma-volume expansion and changes in renal glucose handling—can reduce circulating 1,5-AG and partially decouple it from contemporaneous glycemia, which may explain the weaker performance when measured only at 24–28 weeks [23, 24]. In our cohort, sampling was done in early pregnancy (~ 14 weeks), which may have preserved the 1,5-AG signal. Moreover, our stratified analyses show that accuracy declines in women with overweight/obesity; thus, cohorts enriched for obesity may more readily conclude that 1,5-AG performs poorly.

In contrast, our study highlights the enhanced predictive performance achieved by combining these markers. Our findings revealed a negligible correlation between 1,5-AG and HbA1c, suggesting that these markers capture distinct and complementary aspects of glucose metabolism. This observation aligns with previous studies [19, 25]. Furthermore, our stratified analysis based on pre-pregnancy BMI provides additional insights into how biomarker performance varies by maternal phenotype and emphasizes that the combined model remains effective across both non-overweight/obese and overweight/obese populations. These findings suggest that a dual-marker screening strategy may offer a more universally applicable approach for early GDM risk stratification.

These findings are consistent with recent studies emphasizing the importance of combining biochemical markers to improve the early prediction of GDM [26, 27]. Several studies have demonstrated that the inclusion of markers, such as adiponectin, sex hormone-binding globulin (SHBG), and high-sensitivity C-reactive protein (hs-CRP), in addition to glycemic indices, enhances diagnostic performance [28–30]. The additive predictive value observed by combining 1,5-AG and HbA1c levels suggests that 1,5-AG may serve as a key component in future multi-marker risk models, particularly in populations with predominant post-load glucose abnormalities.

Given the limited sensitivity of current risk factor-based screening strategies [31], the incorporation of early biochemical markers such as 1,5-AG and HbA1c could enhance GDM detection strategies. Although the American College of Obstetricians and Gynecologists does not currently recommend routine screening for GDM before 24 weeks of gestation [6], this position reflects the lack of established methodologies to assess early glycemic abnormalities rather than a dismissal of the associated risks [6, 32, 33]. Currently, RBG is often used for early assessments; however, our findings suggest that 1,5-AG and HbA1c are strongly correlated with fasting and post-load glucose levels. The dual-marker screen (HbA1c + 1,5-AG) in early pregnancy could be used alongside or instead of early RBG to select candidates for confirmatory OGTT. A rule—OGTT if RBG ≥ 100 mg/dL or a pre-specified dual-marker threshold is exceeded—may capture post-prandial dysglycemia missed by RBG alone and enable phenotype-aware triage. Thresholds should be pre-specified and prospectively validated. Regarding feasibility, unit prices in our setting are modest, but costs are context-dependent, so implementation in resource-constrained settings warrants local assessment. By reducing the number of OGTTs, this workflow can decrease patient burden—OGTT typically entails ≥ 3 venipunctures (fasting, 1-h, 2-h) and extended clinic time—while preserving a route to confirmatory testing.

This study has several methodological and contextual strengths. First, it is among the few studies examining the combined use of 1,5-AG and HbA1c in early pregnancy to predict GDM. Although previous studies have evaluated these markers individually with mixed results, our findings demonstrate that combining them significantly improves diagnostic performance. Second, we revealed phenotype-specific differences in biomarker utility by conducting stratified analyses according to pre-pregnancy BMI, highlighting that 1,5-AG performs better in women with lower BMIs, whereas HbA1c is more predictive in those with higher BMIs. This study provides clinically relevant insights that expand upon previous research.

Nonetheless, this study has several limitations. This retrospective study included 187 pregnancies in which 1,5-AG testing was obtained voluntarily. Women who underwent testing may have been at a higher risk of hyperglycemia, either by themselves or by their clinicians. It is therefore plausible that women who perceived themselves to be at higher risk (e.g., family history of diabetes, prior GDM, higher BMI) were more likely to opt in, introducing potential selection bias and potentially contributing to the higher observed GDM prevalence. As our screening workflow and GDM definition used Japan-specific OGTT thresholds, international generalizability to settings applying other criteria may be limited. The generalizability of our findings to lower-risk populations remains uncertain, and our relatively small sample size underscores the need for large-scale studies to evaluate their integration into practical screening algorithms and their potential to improve pregnancy outcomes across diverse clinical settings. Second, pre-pregnancy BMI is based on self-reported weight; thus, measurement error is possible. Third, our dual-marker model and Youden-optimal cut-offs were derived and evaluated in the same dataset without temporal or external/prospective validation; therefore, performance estimates should be considered exploratory and setting-specific. Furthermore, because this was a retrospective analysis based on electronic medical records from two tertiary centers, the findings may not be generalizable to other settings or populations. Therefore, prospective validation in a broader population-based cohort is warranted.

In conclusion, the combination of 1,5-AG and HbA1c improved GDM prediction by capturing complementary glycemic abnormalities linked to BMI-specific metabolic phenotypes, with each marker providing complementary information. Our findings suggest that these biomarkers may complement or provide an early adjunct to existing screening tools. But considering limitations in generalizability due to selection bias, this dual-marker approach warrants further validation.

## Supplementary Information

Below is the link to the electronic supplementary material.

Supplementary file1 (DOCX 41 KB)

Supplementary file2 (DOCX 166 KB)

## Data Availability

The datasets used and/or analyzed during the current study are available from the corresponding author upon reasonable request and with the permission of the ethics board of Nagoya University Hospital.
