# Supplementary material for: Synergistic use of 1,5-AG and HbA1c for early prediction of gestational diabetes: capturing BMI-dependent glycemic phenotypes
Source: Arch Gynecol Obstet. 2026 Jan 2;313(1):4. doi: 10.1007/s00404-025-08281-3 (PMC12764630; doi:10.1007/s00404-025-08281-3)
Supplement: Supplementary file 1 — Supplementary file1 (DOCX 41 KB) [file 404_2025_8281_MOESM1_ESM.docx]

**Figure S1. Study flowchart for participant selection and inclusion criteria.**


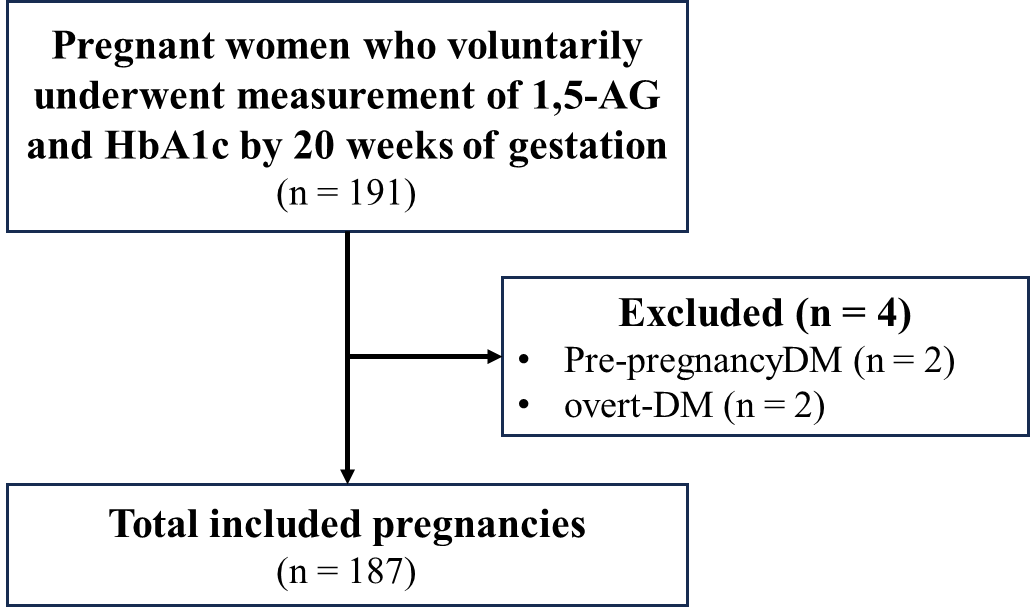


A total of 191 pregnant women who voluntarily underwent measurement of 1,5-anhydroglucitol (1,5-AG) and hemoglobin A1c (HbA1c) by 20 weeks of gestation were initially identified. Four participants were excluded due to pre-existing diabetes (n = 2) or overt diabetes diagnosed during pregnancy (n = 2), resulting in 187 pregnancies included in the final analysis.
