# Supplementary material for: Synergistic use of 1,5-AG and HbA1c for early prediction of gestational diabetes: capturing BMI-dependent glycemic phenotypes
Source: Arch Gynecol Obstet. 2026 Jan 2;313(1):4. doi: 10.1007/s00404-025-08281-3 (PMC12764630; doi:10.1007/s00404-025-08281-3)
Supplement: Supplementary file 2 — Supplementary file2 (DOCX 166 KB) [file 404_2025_8281_MOESM2_ESM.docx]

**Figure S2. Distributions of early-pregnancy biomarkers**


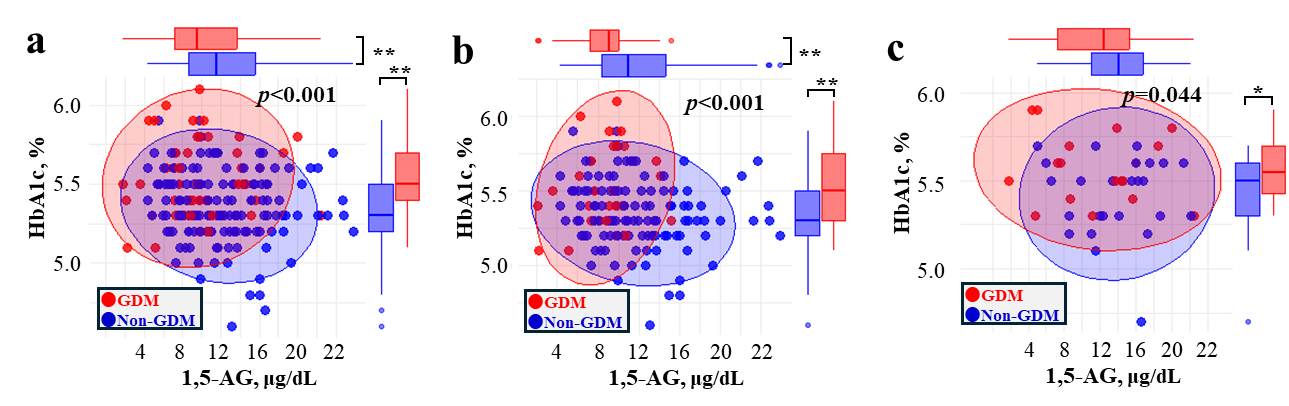


(a): Overall cohort; (b): non-overweight/obesity (BMI < 25.0 kg/m²); (c): Overweight/obesity (BMI ≥ 25.0 kg/m²).

Scatter plots of 1,5-AG vs. HbA1c by GDM status with 95% confidence intervals and marginal box plots (horizontal axis = HbA1c, vertical axis = 1,5-AG). Red: GDM; blue: non-GDM. Boxplots show the median, IQR, and whiskers (1.5 × IQR).

*p* values within the scatterplots are from MANOVA; asterisks on boxplots indicate *p* values from univariate ANOVAs.

**p* < 0.05, ***p* < 0.01.
